# The use of androgen deprivation therapy for prostate cancer and its effect on the subsequent dry eye disease: a population-based cohort study

**DOI:** 10.7150/ijms.73417

**Published:** 2022-06-21

**Authors:** Hsiang-Wen Chien, Chiao-Wen Lin, Chia-Yi Lee, Jing-Yang Huang, Shun-Fa Yang, Kai Wang

**Affiliations:** 1 Department of Ophthalmology, Cathay General Hospital, Taipei, Taiwan.; 2 Departments of Ophthalmology, Sijhih Cathay General Hospital, New Taipei City, Taiwan.; 3 School of Medicine, College of Medicine, Fu Jen Catholic University, New Taipei, Taiwan.; 4 School of Medicine, National Tsing Hua University, Hsinchu, Taiwan, Taiwan.; 5 Institute of Oral Sciences, Chung Shan Medical University, Taichung, Taiwan.; 6 Department of Dentistry, Chung Shan Medical University Hospital, Taichung, Taiwan.; 7 Department of Ophthalmology, Nobel Eye Institute, Taipei, Taiwan.; 8 Institute of Medicine, Chung Shan Medical University, Taichung, Taiwan.; 9 Department of Medical Research, Chung Shan Medical University Hospital, Taichung, Taiwan.

**Keywords:** androgen deprivation therapy, dry eye disease, age, database, epidemiology

## Abstract

This study aimed to investigate the influence of androgen deprivation therapy (ADT) for the development of dry eye disease (DED) in subjects with prostate cancer via the use of national health insurance research database (NHIRD) of Taiwan. A retrospective cohort study was conducted and patients were selected as prostate cancer with ADT according to diagnostic and procedure codes. Each participant in that group was then matched to one patient with prostate cancer but without ADT and two subject s without prostate cancer and ADT. And a total of 1791, 1791 and 3582 participants were enrolled in each group. The primary outcome was set as the DED development according to the diagnostic codes. Cox proportional hazard regression was applied to calculate the adjusted hazard ratio (aHR) and 95% confidence interval (CI) of ADT and other parameters for DED development. There were 228, 126 and 95 new events of DED developed in the control group, the prostate cancer without ADT group and the prostate cancer with ADT group. The rate of DED in the prostate cancer with ADT group (aHR: 0.980, 95% CI: 0.771-1.246, P= 0.8696) and Prostate cancer without ADT group (aHR: 1.064, 95% CI: 0.855-1.325, P= 0.5766) were not significantly different compared to the control group. In addition, the patients aged 70-79 years old demonstrated a significantly higher incidence of developing DED compared to those aged 50-59 years old (aHR: 1.885, 95% CI: 1.188-2.989, P= 0.0071). In conclusion, the use of ADT did not alter the incidence of subsequent DED.

## Introduction

The prostate cancer is a prevalent cancer in male population 1, with more than 1,400,000 new cases of prostate cancer and 370,000 related deaths were reported in 2020 globally 2. About the treatment of ADT, the androgen deprivation therapy (ADT) has been used as a common therapy that can reduce the prostate function and suppress the progression of prostate cancer 1, 3, 4. The treatment options of ADT in prostate cancer include the LHRH agonists, estrogens, antiandrogens, and orchiectomy 5. The median survival duration for prostate cancer was about 14 years under the ADT management 6, and the early use of ADT showed certain benefits for patients with prostate cancer and nodal metastases 7.

Several complications had been reported after the ADT management 8. The cardiovascular disorders are common complications after the ADT arrangement 8, 9. According to one research, the subjects received ADT were correlated to higher incidence of ischemic stroke and coronary arterial diseases 8. Besides, the rate of sudden cardiac death was significantly higher in patients received the ADT 10. In addition to the above disorders, the ADT is associated with the development of deep vein thrombosis 11. There were some other complications after ADT which include the cognitive decline, anemia, osteoporosis, depression and diabetes mellitus (DM) 12-14.

The hormone status, like the level of growth factor and estrogen, are known to influence the ocular condition 15, 16. The dry eye disease (DED) is a multifactorial disorder that features with tear film dysfunction and ocular surface damage 17. According to previous experience, the aromatase inhibitor therapy would result in DED symptoms 18, and the use of 5α-Reductase inhibitor finasteride would also contribute to androgen deficiency DED 19. About other experiences between androgen deficiency status and DED, one study demonstrated the protective effect of androgen on DED while another randomized controlled double-masked study showed insignificant correlation between the androgen level and DED development 20, 21. Consequently, additional long-term research may be conduct to survey this issue more clearly.

The purpose of the current study is to investigate the possible relationship between the ADT and subsequent DED via the application of the national health insurance research database (NHIRD) of Taiwan. In addition to ADT, other potential risk factors for DED occurrence were also evaluated in the statistical analysis.

## Materials and Methods

### Data source

Our retrospective cohort study adhered to the declaration of Helsinki in 1964 and its later amendment, and the current study was approved by both the Institutional Review Board of Chung Shan Medical University (Project identification code: CS1-20108), and the National Health Insurance Administration. Moreover, the need of informed consent from subjects was waived by the two institutions. NHIRD of Taiwan contains the claimed data of health insurance service for nearly all Taiwanese that means about 23 million individuals. The interval of NHIRD ranged from January 1, 2000 till December 31, 2018, and the data available from NHIRD include the International Classification of Diseases, Ninth Revision (ICD-9) diagnostic code, International Classification of Diseases, Tenth Revision (ICD-10) diagnostic codes, demographic data, examination code, code of procedure and international ATC codes for all medications. In our study, we used the longitudinal health insurance database (LHID) 2005 version, which is one of the sub-databases from NHIRD, for all the analyses. In LHID 2005, approximately two million patients were randomly selected from the NHIRD at the year of 2005, and these individuals were followed as the same time period as in the NHIRD.

### Patient Selection

Men aged from 40 to 100-year-old who received ICD-9 or ICD-10 diagnostic codes of prostate cancer and experienced aromatase inhibitors, LHRH agonists, antiandrogens, estrogens or bilateral orchiectomy (according to procedure/ATC codes) were included in the prostate cancer with ADT group. The exclusion criteria included blindness, ocular tumor, eyeball removal procedure, severe ocular trauma, DED development or death before index date, ADT prior to prostate cancer diagnosis and prostate cancer developed before 2001 (n=572). The index date was defined as six months after the starting of ADT. Then each subject with prostate cancer and ADT was matched to one prostate cancer participant without ADT and two non-prostate cancer patients. If a prostate cancer patient with ADT cannot be matched to individuals in other two populations, that person would be discarded. The match method is propensity-score matching (PSM) with age and socio-economic status, and the non-prostate cancer population constituted the control group. In our study, 1,791, 1,791 and 3,582 patients were enrolled in the prostate cancer with ADT group, prostate cancer without ADT group and the control groups.

### Main Outcome Measurement

The primary outcome is the development of DED which defined as (1) the diagnosis of DED based on the corresponded ICD-9 and ICD-10 diagnostic codes, (2) the arrangement of fluorescein test or Schirmer's test before the diagnosis of DED, and (3) the DED was diagnosed by an ophthalmologist. To survey the possible correlation between the ADT and DED, only the DED developed after the index date was defined as the achievement of the primary outcome in the current study.

### Demographic and Co-morbidity Variables

To let the general status of our study population more homogenous, the effects of the following parameters were included in the multivariable analysis: age, urbanization, occupation, hypertension, diabetes mellitus (DM), coronary arterial disease (CAD), acute myocardial infarction (AMI), hyperlipidemia, cerebrovascular disease and dementia. The existence of these parameters was according to related ICD-9 and ICD-10 diagnostic codes for all the diseases. Besides, the CAD referred to those with chronic ischemic heart disease according to ICD-9 and ICD-10 diagnostic codes. All participants were followed longitudinally since the index date to the date of DED diagnosis, quit from the National Health Insurance program, or the end of NHIRD interval, which also known as the 31 December, 2018.

### Statistical Analysis

SAS version 9.4 (SAS Institute Inc, NC, USA) was used for all the statistical analyses. After the PSM method, we used descriptive analysis to show the baseline characters of the three groups. The Poisson regression was used for the incidence rate of DED with corresponding 95% confidence interval (CI) among the groups. Then Cox proportional hazard regression was applied to estimate the crude as well as the adjusted hazard ratio (aHR) of DED among the three groups which considered the possible effects of the demographic data and systemic diseases in our multivariable analysis. Besides, Cox proportional hazard regression was also used to evaluate the effect of each parameter on the development of DED and presented as aHR with 95% CI. In the next step, we made the Kaplan-Meier curves to illustrate the cumulative probability of DED among the prostate cancer with ADT group, prostate cancer without ADT group and the control group, then the log rank test was used to investigate whether significant difference exist among the three survival curves from different groups. The threshold of statistical significance was set at P < 0.05.

## Results

The baseline characters of the study population are shown in Table 1. The distribution of age, urbanization and occupation were similar among the three groups due to PSM process. Moreover, the rate of systemic co-morbidities were also statistical insignificant among the three groups although a numerically higher rate of systemic diseases was found in the prostate cancer with ADT group. For the type of ADT, the antiandrogens therapy was the most commonly used ADT which 67.67 percent of patients received such management, while 61.86 percent, 11.28 percent and 7.82 percent of subjects received LHRH agonists, bilateral orchiectomy and estrogen therapy, respectively (Table 1).

**Figure 1:**
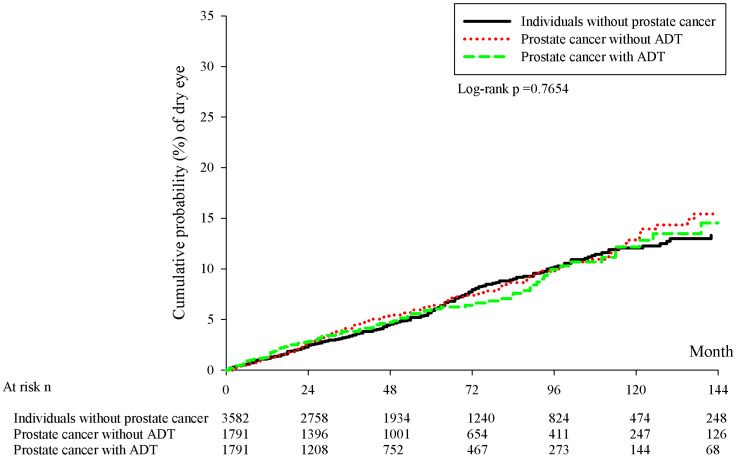
Kaplan-Meier curves with cumulative probability of dry eye disease among the three groups. ADT: androgen deprivation therapy; DED: dry eye disease; n: number.

There were 228, 126 and 95 new cases of DED occurred in the control group, the prostate cancer without ADT group and the prostate cancer with ADT group, respectively. In the Cox regression analysis, the incidence of DED in the prostate cancer with ADT group (aHR: 0.980, 95% CI: 0.771-1.246, P= 0.8696) and Prostate cancer without ADT group (aHR: 1.064, 95% CI: 0.855-1.325, P= 0.5766) were not significantly different compared to the control group (Table 2). Besides, the cumulative probabilities of DED development were similar among the three groups at different time point (P= 0.1413) (Figure 1).

In the analysis of different parameters, the patients aged 70-79 years old showed a significantly higher risk of developing DED compared to those aged 50-59 years old (aHR: 1.885, 95% CI: 1.188-2.989, P= 0.0071). The other parameters, including the demographic data and systemic disorders, did not demonstrated significant influence on the occurrence of DED (all P> 0.05) (Table 3).

## Discussion

Briefly, the current study showed the insignificant effect of ADT on the development of DED in patients with prostate cancer. In addition, the cumulative probability of DED among different patient groups did not reveal significant difference with time. On the other hand, the age between 70 to 79 years old demonstrated a prominent influence on the development of DED which served as an independent risk factor.

The formation DED is thought to be multifactorial while the inflammatory reaction is the major mechanism according to the literatures conducted recently 17, 22, 23. In the report published by the Dry Eye Workshop, the development of DED is due to the vicious cycle the damage the ocular surface 22. As the tear film became instable, the osmorlarity of the tear film would increase which can be exaggerated by the presence of meibomian gland dysfunction 22. Then the inflammatory cytokine like the interleukin and tumor necrosis factors were released and cause damage to the goblet cell as well as corneal epithelium, resulting in unstable tear film 22. Consequently, the disorder that could induce inflammatory reaction owns the chance to elevate the risk of DED development 24. Some autoimmune diseases were associated with the DED occurrence in previous studies, which included the Sjogren syndrome, rheumatoid arthritis, systemic lupus erythematous and gout arthritis 25-28. On the other hand, the change of hormone status can also lead to the production of inflammation cytokine 29. In previous study, the estrogen is associated with the elevation of interleukins and reactive oxygen species 30. Besides, the relationship between androgen and the suppression of inflammatory reaction had been established 31, 32. However, there was no strong correlation between the androgen deficiency and the autoimmune disease, which indicated that the elevation of inflammatory process is not always cause inflammation-related disease. Moreover, the androgen deficiency status did not cause lacrimal gland inflammation in experimental study 33. Since DED is correlated to several inflammatory processes and ADT could alter the inflammation reaction 19, 24, the potential effect of ADT on DED development should be surveyed while the results of the current study demonstrated an insignificant association between the ADT and DED.

The relationship between ADT and DED has not been established firmly in previous researches 19-21, 34, while the result of the current study illustrated a minimal influence of ADT on the subsequent DED. About the two studies that showed a significant effect of ADT on DED, one was experimental studies which used DED model to survey the potential relationship between androgen deficiency and DED 19. Another prospective study that supported the association between DED and androgen recruited only 50 participants, and they concluded that the application of androgen transdermal device can decrease the severity of DED 21. In the current study, we enrolled approximately 7 thousands participants in the whole study population and the follow up period can up to 18 years. Furthermore, the current study enrolled multiple parameters in the analysis model to erase the effect of possible confounders thus the results may be more reliable compared to the researches that evaluate the relationship between androgen deficiency and DED but without considering the influence of other factors 19, 21. On the other hand, the cumulative probability of DED in the prostate cancer with ADT group did not elevate throughout the study interval compared to the prostate cancer without ADT group and the control group, which may indicates the long-term application of ADT did not increase the incidence of DED compared to non-ADT user.

Concerning the other parameters that may contribute to the development of DED, the age range from 70 to 79 years old showed a significantly higher rate of DED occurrence compared to those aged 50 to 59 years old. The age is a well-established risk factor for DED development 35. And about the parameters of DED, older age is correlated to shorter tear break-up time and ocular surface stains compared to younger individuals 36. In the current study, the significant correlation of old age to DED development compared to the younger population was compatible to previous experience. However, the patients aged 80 years or older did not reveal significantly higher incidence of DED compared to those aged 50 to 59 years old. There are two possible explanations for the conflicting results. Firstly, the patients older than 80 years old may become more disable and thus would not visit the ophthalmic department as easy as their younger counterpart 37, thus the diagnostic rate of DED could be reduced. Another possible reason is because the visual display terminal is another prominent risk factors for DED 38, and patients aged more than 80 years old might not use these device commonly according to clinical experience. The other parameters did not show significant effect of the development of DED. Although DM was associated with impaired corneal epithelial wound healing 39, the influence of this corneal injury may not induce persistent ocular inflammation and following DED.

About the epidemiology aspect, the DED is a prevalent disease in the elderly population 35. In an epidemiological research, the prevalence of DED was about 11.3 percent in the population older than 50 years 40. Although the female is more vulnerable to the DED, the prevalence of DED in the male population still reached 5.65 percent in that study.^40^ On the other hand, the prostate cancer is one of the most common cancers in the elderly male population 41, 42. According to a previous research, the prevalence of prostate cancer is above 30 per 100000 male in Asian region 43. Moreover, the ADT was applied in nearly all the prostate cancer individuals 1. Because both DED and prostate cancer affect a majority of elderly male population and ADT is widely applied in those with prostate cancer 1, 40, the importance to investigate whether ADT is related to following DED occurrence cannot be overemphasized.

There are some limitations in the current study. First, the retrospective design of the current study and the nature of claimed-data research will diminish the homogeneity and the accuracy of the current study. Second, we can only know the patient received DED-related exams and ADT, while the severity and treatment outcome of both prostate cancer and DED cannot be obtained in the NHIRD/LHID. Besides, we did not analyze the effect of different ADT on DED separately because many participants in the current study received more than one type of ADT. Also, more than half of patients with prostate cancer and received ADT management were excluded in the matching process which may reduce the statistical power. Nevertheless, since we want to ensure the homogeneity among different groups and the case numbers in the current study is not inferior to previous studies that survey the ADT 14, 44, the influence of this limitation may not be prominent.

In conclusion, the application of ADT did not cause higher incidence of subsequent DED either in short-term or long-term utilization. Furthermore, old age is still a risk factor for DED development especially in those aged 70-79 years old. Consequently, the use of ADT may be safe even in those with predisposing factors for DED. Further large-scale prospective study that evaluates whether the use of ADT will affect the therapeutic outcome of DED is mandatory.

## Figures and Tables

**Table 1:** Baseline characteristics among study population

Character	Control (n= 3582)	Prostate cancer without ADT (n= 1791)	Prostate cancer with ADT (n= 1791)	P value
**Age at index**				0.9607
<50	19 (0.53%)	9 (0.50%)	10 (0.56%)	
50-59	220 (6.14%)	101 (5.64%)	107 (5.97%)	
60-69	922 (25.74%)	453 (25.29%)	463 (25.85%)	
70-79	1498 (41.82%)	778 (43.44%)	737 (41.15%)	
≥80	923 (25.77%)	450 (25.13%)	474 (26.47%)	
**Urbanization**				0.8220
Urban	2017 (56.31%)	995 (55.56%)	982 (54.83%)	
Sub-urban	1160 (32.38%)	580 (32.38%)	596 (33.28%)	
Rural	405 (11.31%)	216 (12.06%)	213 (11.89%)	
**Occupation**				0.7806
Government employees	279 (7.79%)	138 (7.71%)	139 (7.76%)	
Labor	1336 (37.30%)	661 (36.91%)	657 (36.68%)	
Farmer and Fisherman	1047 (29.23%)	553 (30.88%)	529 (29.54%)	
Low income	13 (0.36%)	13 (0.73%)	12 (0.67%)	
Unemployed	855 (23.87%)	401 (22.39%)	428 (23.90%)	
Others	52 (1.45%)	25 (1.40%)	26 (1.45%)	
**Co-morbidities**				
Hypertension	1907 (53.24%)	951 (53.10%)	961 (53.66%)	0.9389
DM	637 (17.78%)	336 (18.76%)	360 (20.10%)	0.1182
CAD	567 (15.83%)	287 (16.02%)	316 (17.64%)	0.2185
AMI	17 (0.47%)	10 (0.56%)	13 (0.73%)	0.5072
Hyperlipidemia	616 (17.20%)	291 (16.25%)	326 (18.20%)	0.3010
Cerebrovascular disease	430 (12.00%)	227 (12.67%)	238 (13.29%)	0.3920
Dementia	91 (2.54%)	47 (2.62%)	56 (3.13%)	0.4446
**ADT type**				
LHRH Agonists			1108 (61.86%)	N/A
Antiandrogens			1212 (67.67%)	N/A
Estrogens			140 (7.82%)	N/A
Bilateral orchiectomy			202 (11.28%)	N/A

ADT: androgen deprivation therapy, DM: diabetes mellitus, CAD: coronary arterial disease, AMI: acute myocardial infarction, N/A: not applicable.

**Table 2:** Incidence risk of study event among study groups

Events	Control	Prostate cancer without ADT	Prostate cancer with ADT
Follow up person months	223473	113755	90335
New case	228	126	95
Incidence rate^#^ (95% CI)	10.20 (8.96-11.62)	11.08 (9.30-13.19)	10.52 (8.60-12.86)
Crude Relative risk (95% CI)	Reference	1.084 (0.872-1.348)	1.024 (0.806-1.301)
aHR (95% CI)	Reference	1.064 (0.855-1.325)	0.980 (0.771-1.246)

^#^ Incidence rate, per 10000 person-months. ADT: androgen deprivation therapy, CI: confidence interval, aHR: adjusted hazard ratio.

**Table 3:** Adjusted hazard ratio of dry eye disease from each parameter

Parameter	aHR	95% CI	P value
**Group**			
Control	Reference		
Prostate cancer without ADT	1.064	0.855-1.325	0.5766
Prostate cancer with ADT	0.980	0.771-1.246	0.8696
**Age at index**			
<50	1.389	0.412-4.684	0.5967
50-59	Reference		
60-69	1.540	0.963-2.462	0.0713
70-79	1.885	1.188-2.989	0.0071*
≥80	1.329	0.798-2.215	0.2743
**Urbanization**			
Urban	Reference		
Sub-urban	1.299	0.941-1.622	0.2070
Rural	1.029	0.703-1.508	0.8820
**Occupation**			
Government employees	0.925	0.638-1.342	0.6829
Labor	Reference		
Farmer and fisherman	0.847	0.630-1.138	0.2705
Low income	1.091	0.259-4.595	0.9058
Unemployed	1.032	0.799-1.335	0.8075
Others	1.063	0.455-2.483	0.8877
**Co-morbidities**			
Hypertension	1.074	0.877-1.316	0.4888
DM	1.117	0.872-1.432	0.3805
CAD	1.129	0.881-1.448	0.3378
AMI	1.092	0.268-4.455	0.9022
Hyperlipidemia	1.280	0.995-1.648	0.0550
Cerebrovascular disease	0.949	0.700-1.286	0.7343
Dementia	0.274	0.068-1.111	0.0699

ADT: androgen deprivation therapy, DM: diabetes mellitus, CAD: coronary arterial disease, AMI: acute myocardial infarction, aHR: adjusted hazard ratio, CI: confidence interval. * denotes significant correlation to dry eye disease development.

## References

[1] Gamat M, McNeel DG (2017). Androgen deprivation and immunotherapy for the treatment of prostate cancer. Endocr Relat Cancer.

[2] Wang L, Lu B, He M, Wang Y, Wang Z, Du L (2022). Prostate Cancer Incidence and Mortality: Global Status and Temporal Trends in 89 Countries From 2000 to 2019. Front Public Health.

[3] Leal F, Figueiredo MA, Sasse AD (2015). Optimal duration of androgen deprivation therapy following radiation therapy in intermediate- or high-risk nonmetastatic prostate cancer: A systematic review and metaanalysis. Int Braz J Urol.

[4] Gourdin T (2020). Recent progress in treating advanced prostate cancer. Curr Opin Oncol.

[5] Crawford ED, Heidenreich A, Lawrentschuk N, Tombal B, Pompeo ACL, Mendoza-Valdes A (2019). Androgen-targeted therapy in men with prostate cancer: evolving practice and future considerations. Prostate Cancer Prostatic Dis.

[6] Harris WP, Mostaghel EA, Nelson PS, Montgomery B (2009). Androgen deprivation therapy: progress in understanding mechanisms of resistance and optimizing androgen depletion. Nat Clin Pract Urol.

[7] Messing EM, Manola J, Yao J, Kiernan M, Crawford D, Wilding G (2006). Immediate versus deferred androgen deprivation treatment in patients with node-positive prostate cancer after radical prostatectomy and pelvic lymphadenectomy. Lancet Oncol.

[8] Gruca D, Bacher P, Tunn U (2012). Safety and tolerability of intermittent androgen deprivation therapy: a literature review. Int J Urol.

[9] Melloni C, Roe MT (2020). Androgen deprivation therapy and cardiovascular disease. Urol Oncol.

[10] Keating NL, O'Malley AJ, Smith MR (2006). Diabetes and cardiovascular disease during androgen deprivation therapy for prostate cancer. J Clin Oncol.

[11] Klil-Drori AJ, Yin H, Tagalakis V, Aprikian A, Azoulay L (2016). Androgen Deprivation Therapy for Prostate Cancer and the Risk of Venous Thromboembolism. Eur Urol.

[12] Andela CD, Matte R, Jazet IM, Zonneveld WC, Schoones JW, Meinders AE (2021). Effect of androgen deprivation therapy on cognitive functioning in men with prostate cancer: A systematic review. Int J Urol.

[13] Izard JP, Siemens DR (2020). Androgen Deprivation Therapy and Mental Health: Impact on Depression and Cognition. Eur Urol Focus.

[14] Chen YZ, Chiang PK, Lin WR, Chen M, Chow YC, Chiu AW (2020). The relationship between androgen deprivation therapy and depression symptoms in patients with prostate cancer. Aging Male.

[15] Peck T, Olsakovsky L, Aggarwal S (2017). Dry Eye Syndrome in Menopause and Perimenopausal Age Group. J Midlife Health.

[16] McKay TB, Priyadarsini S, Karamichos D (2022). Sex Hormones, Growth Hormone, and the Cornea. Cells.

[17] Milner MS, Beckman KA, Luchs JI, Allen QB, Awdeh RM, Berdahl J (2017). Dysfunctional tear syndrome: dry eye disease and associated tear film disorders - new strategies for diagnosis and treatment. Curr Opin Ophthalmol.

[18] Serban D, Costea DO, Zgura A, Tudosie MS, Dascalu AM, Gangura GA (2022). Ocular Side Effects of Aromatase Inhibitor Endocrine Therapy in Breast Cancer - A Review. *In vivo*.

[19] Li K, Zhang C, Yang Z, Wang Y, Si H (2017). Evaluation of a novel dry eye model induced by oral administration of finasteride. Mol Med Rep.

[20] Azcarate PM, Venincasa VD, Feuer W, Stanczyk F, Schally AV, Galor A (2014). Androgen deficiency and dry eye syndrome in the aging male. Invest Ophthalmol Vis Sci.

[21] Supalaset S, Tananuvat N, Pongsatha S, Chaidaroon W, Ausayakhun S (2019). A Randomized Controlled Double-Masked Study of Transdermal Androgen in Dry Eye Patients Associated With Androgen Deficiency. Am J Ophthalmol.

[22] Bron AJ, de Paiva CS, Chauhan SK, Bonini S, Gabison EE, Jain S (2017). TFOS DEWS II pathophysiology report. Ocul Surf.

[23] Thulasi P, Djalilian AR (2017). Update in Current Diagnostics and Therapeutics of Dry Eye Disease. Ophthalmology.

[24] Yamaguchi T (2018). Inflammatory Response in Dry Eye. Invest Ophthalmol Vis Sci.

[25] Lee CY, Chen HC, Sun CC, Lin HY, Lu KH, Huang JY (2019). Gout as a Risk Factor for Dry Eye Disease: A Population-Based Cohort Study. J Clin Med.

[26] Abd-Allah NM, Hassan AA, Omar G, Hamdy M, Abdelaziz STA, Abd El Hamid WM (2020). Dry eye in rheumatoid arthritis: relation to disease activity. Immunol Med.

[27] Wang L, Xie Y, Deng Y (2021). Prevalence of dry eye in patients with systemic lupus erythematosus: a meta-analysis. BMJ Open.

[28] Perez VL, Stern ME, Pflugfelder SC (2020). Inflammatory basis for dry eye disease flares. Exp Eye Res.

[29] Kovats S (2015). Estrogen receptors regulate innate immune cells and signaling pathways. Cell Immunol.

[30] Roy D, Cai Q, Felty Q, Narayan S (2007). Estrogen-induced generation of reactive oxygen and nitrogen species, gene damage, and estrogen-dependent cancers. J Toxicol Environ Health B Crit Rev.

[31] Blanquart E, Mandonnet A, Mars M, Cenac C, Anesi N, Mercier P (2022). Targeting androgen signaling in ILC2s protects from IL-33-driven lung inflammation, independently of KLRG1. J Allergy Clin Immunol.

[32] Gandhi VD, Cephus JY, Norlander AE, Chowdhury NU, Zhang J, Ceneviva ZJ (2022). Androgen receptor signaling promotes Treg suppressive function during allergic airway inflammation. J Clin Invest.

[33] Sullivan DA, Krenzer KL, Sullivan BD, Tolls DB, Toda I, Dana MR (1999). Does androgen insufficiency cause lacrimal gland inflammation and aqueous tear deficiency?. Invest Ophthalmol Vis Sci.

[34] Wang L, Deng Y (2020). The applications of androgen in the treatment of dry eye disease: a systematic review of clinical studies. Endocr J.

[35] Moss SE, Klein R, Klein BE (2008). Long-term incidence of dry eye in an older population. Optom Vis Sci.

[36] Hashemi H, Khabazkhoob M, Kheirkhah A, Emamian MH, Mehravaran S, Shariati M (2014). Prevalence of dry eye syndrome in an adult population. Clin Exp Ophthalmol.

[37] Wang X, Sun M, Li X, Lu J, Chen G (2020). Effects of Disability Type on the Association between Age and Non-Communicable Disease Risk Factors among Elderly Persons with Disabilities in Shanghai, China. Int J Environ Res Public Health.

[38] Fjaervoll H, Fjaervoll K, Magno M, Moschowits E, Vehof J, Dartt DA (2022). The association between visual display terminal use and dry eye: a review. Acta Ophthalmol.

[39] Shih KC, Lam KS, Tong L (2017). A systematic review on the impact of diabetes mellitus on the ocular surface. Nutr Diabetes.

[40] Farrand KF, Fridman M, Stillman I, Schaumberg DA (2017). Prevalence of Diagnosed Dry Eye Disease in the United States Among Adults Aged 18 Years and Older. Am J Ophthalmol.

[41] Chou YE, Hsieh MJ, Wang SS, Lin CY, Chen YY, Ho YC (2021). The impact of receptor of advanced glycation end-products polymorphisms on prostate cancer progression and clinicopathological characteristics. J Cell Mol Med.

[42] Hu JC, Wang SS, Chou YE, Chiu KY, Li JR, Chen CS (2021). Associations between LncRNA MALAT1 Polymorphisms and Lymph Node Metastasis in Prostate Cancer. Diagnostics (Basel).

[43] Kimura T, Egawa S (2018). Epidemiology of prostate cancer in Asian countries. Int J Urol.

[44] Harrington JM, Schwenke DC, Epstein DR, Bailey DE Jr (2014). Androgen-deprivation therapy and metabolic syndrome in men with prostate cancer. Oncol Nurs Forum.

